# Impact of resection margin on outcome in soft-tissue sarcomas of the extremities treated with limb-sparing surgery and postoperative radiotherapy

**DOI:** 10.1186/s12957-024-03380-y

**Published:** 2024-04-26

**Authors:** Chun-Chieh Chen, Yao-Yu Wu, Jo-Ting Kao, Chih‑Hsiang Chang, Shih-Chiang Huang, Hsin‑Nung Shih

**Affiliations:** 1 Department of Orthopedic Surgery, Chang Gung Memorial Hospital, Linkou, No. 5, Fuxing Street, Guishan District Taoyuan City, 333 Taiwan; 2 Department of Radiation Oncology, Chang Gung Memorial Hospital, Keelung, No. 222, Maijin Rd., Anle Dist Keelung City, 204 Taiwan; 3 Department of Anatomic Pathology, Chang Gung Memorial Hospital, Linkou, No. 5, Fuxing Street, Guishan District Taoyuan City, 333 Taiwan; 4 College of Medicine, Chang Gung University, No. 259, Wenhua 1 Road, Guishan District Taoyuan City, 333 Taiwan; 5 Bone and Joint Research Center, Chang Gung Memorial Hospital, Linkou, No. 5, Fuxing Street, Guishan District Taoyuan City, 333 Taiwan; 6 Hejiang Orthopedic Clinic, No. 200, Zhongzheng E. Rd., Zhubei City, Hsinchu County 302 Taiwan

**Keywords:** FNCLCC grade, Microscopically negative margin (R0), Microscopically positive margin (R1), Recurrence, Soft-tissue sarcomas

## Abstract

**Background:**

The standard curative treatments for extremity soft tissue sarcoma (ESTS) include surgical resection with negative margins and perioperative radiotherapy. However, the optimal resection margin remains controversial. This study aimed to evaluate the outcomes in ESTS between microscopically positive margin (R1) and microscopically negative margin (R0) according to the Union for International Cancer Control (UICC) (R + 1 mm) classification.

**Methods:**

Medical records of patients with localized ESTS who underwent primary limb-sparing surgery and postoperative radiotherapy between 2004 and 2015 were retrospectively reviewed. Patients were followed for at least 5 years or till local or distant recurrence was diagnosed during follow-up. Outcomes were local and distal recurrences and survival.

**Results:**

A total of 52 patients were included in this study, in which 17 underwent R0 resection and 35 underwent R1 resection. No significant differences were observed in rates of local recurrence (11.4% vs. 35.3%, *p* = 0.062) or distant recurrence (40.0% vs. 41.18%, *p* = 0.935) between R0 and R1 groups. Multivariate analysis showed that distant recurrences was associated with a Fédération Nationale des Centres de Lutte Contre le Cancer (FNCLCC) grade (Grade III vs. I, adjusted hazard ratio (aHR): 12.53, 95% confidence interval (CI): 2.67–58.88, *p* = 0.001) and tumor location (lower vs. upper extremity, aHR: 0.23, 95% CI: 0.07–0.7, *p* = 0.01). Kaplan–Meier plots showed no significant differences in local (*p* = 0.444) or distant recurrent-free survival (*p* = 0.161) between R0 and R1 groups.

**Conclusions:**

R1 margins, when complemented by radiotherapy, did not significantly alter outcomes of ESTS as R0 margins. Further studies with more histopathological types and larger cohorts are necessary to highlight the path forward.

## Background

Soft tissue sarcomas (STS) are rare tumors of mesenchymal origin, accounting for approximately 1% of all adult malignancies in the United States in 2021 [1]. The standard treatment of primary localized extremity STS (ESTS) includes limb-sparing surgical resection with negative margins alone or combined with perioperative radiotherapy and/or chemotherapy to prevent local recurrence with maintaining optimal function [2, 3]. Adjuvant radiotherapy has been reported to effectively prevent local recurrence in STS [4].

The optimal resection margin to prevent local recurrence still remains controversies in the clinical practice. Many studies showed that a negative margin is essential for achieving the best local control [5–9]. The tumors should be resected with a sufficiently wide margin of the surrounding normal tissue, because a positive surgical margin increases the risk of local recurrence, even with radiotherapy [4, 10]. However, some studies showed that positive resection margin achieved good local control and survival as negative margin and avoided a radical approach [11–14]. Considering the maximal postoperative function and minimal morbidity, sometimes a negative surgical margin is hard to achieve if tumors are near uninvolved critical neurovascular structures, bones, and joints. Therefore, this retrospective study aimed to assess the impact of resection margin on outcome in ESTS treated with limb-sparing surgery and postoperative radiotherapy between microscopically negative resection (R0) and microscopically positive resection (R1).

## Materials and Methods

### Study design

We retrospectively reviewed medical records of patients diagnosed with localized ESTS from the cancer registration database of Chang Gung Memorial Hospital using International Classification of Diseases, Ninth Revision and Tenth Revision, Clinical Modification (ICD-9-CM, ICD-10-CM) codes (ICD-9: 171.2, 171.3; ICD-10: C47.10, C47.11, C47.12, C49.10, C49.11, C49.12, C47.20, C47.21, C47.22, C49.20, C49.21, C49.22). Inclusion criteria were: 1) Age > 18 years old; 2) Underwent primary limb-sparing surgery with R0 or R1 resection and postoperative radiotherapy at our institution between 2004 and 2015; 3) No distant metastasis in primary diagnosis; 4) Follow-up ≧ 5 years, or the local/distant recurrence diagnosed within 5 years after the primary surgery. Exclusion criteria were: 1) Patients received the definitive surgical resection at other institutions; 2) Had metastatic disease diagnosed at the time of primary diagnosis; 3) Underwent an incomplete (R2) resection; 4) Did not receive postoperative radiotherapy; 5) Lost follow-up. Resection margin was defined according to the Union for International Cancer Control (UICC) (R + 1 mm classification) [15]. R1 was defined if the resection margin to tumor was within 1 mm of the inked border, and R0 was with at least 1 mm of normal tissue between the tumor and the inked resection margin. Patients were followed up for at least 5 years or till local or distant recurrence diagnosed during follow-up. The study protocol was reviewed and approved by the Institutional Review Board, and ethics committee specifically waived the requirement for informed consent.

Patients’ demographic information and tumor profiles were collected from medical charts. Histologic diagnosis, tumor grade dependent on a Fédération Nationale des Centres de Lutte Contre le Cancer (FNCLCC) grading [16] system, and tumor size were obtained from the pathology reports evaluated by pathologists with expertise in soft tissue sarcomas. Patients were regularly followed at 1 month and 3 months after the primary surgery, then every 3 months for the next 3 years, every 6 months for the 4th and 5th years, and then yearly after that, typically until year 10. During follow-up period, each patient received computed tomography of the chest to monitor the presence of pulmonary metastases and magnetic resonance imaging of the primary site to detect the presence of local recurrent annually, or when needed.

### Radiotherapy

Postoperative radiotherapy was delivered through a 6MV linear accelerator to the target volume by three-dimensional conformal radiotherapy (3DCRT), intensity modulated radiotherapy (IMRT), or volumetric modulated arc therapy (VMAT), in a schedule of 2 Gy/fraction, five fractions per week. An initial dose of 50 Gy was delivered after surgical wound healing (generally between 6–8 weeks after surgery) with the target volume to the surgical bed, which was expanded with a 1.5 cm radial margin and a 4–5 cm cranio-caudal margin to encompass microscopic disease in the surrounding tissues; the last boost dose of 10–20 Gy was applied to the original tumor bed with a 1–1.5 cm radial margin and a 2–3 cm cranio-caudal margin.

### Endpoints

The primary endpoints were local recurrence and distant recurrence. Local recurrence was defined as any tumor recurrence at the primary tumor site, and distant recurrence was defined as all other tumor recurrences. Local recurrence-free survival was calculated from the date of the surgery to the date of first local recurrence diagnosed or censored at the date of last follow-up assessment in local recurrence-free patients. Distant recurrence-free survival was calculated from the date of the surgery to the date of first distant recurrence diagnosed or censored at the date of last follow-up assessment in distant recurrence-free patients.

### Statistical analysis

Descriptive statistics of patients’ demographic and clinical characteristics are presented as number (n) and percentage (%) and performed by the chi-squared test or Fisher Exact test. Continuous data with normal distribution are presented as mean ± standard deviation (SD) using Student’s test. Hazard ratios (HRs) and 95% confidence intervals (CIs) produced by Cox regression analysis were used to evaluate associations between covariates and event occurrence. Any variable whose univariate analysis had a *p*-value < 0.15 was input as a candidate for the multivariable analysis. Kaplan Meier plot and log-rank test were conducted to compare the patients with ESTS underwent postoperative radiotherapy between R1 and R0. The results were considered statistically significant at *p* < 0.05, and all statistical analyses were performed using the statistical package SPSS for Windows (Version 21.0, SPSS Inc., IBM Corp., Armonk, NY, USA).

## Results

### Baseline characteristics

Patients’ baseline characteristics and tumor profiles are presented in Table 1. A total of 52 patients were included in this study, in which 35 patients underwent R1 and 17 patients underwent R0. Patients’ mean age was 51.0 ± 18.7 years and 53.8% were male. The mean follow-up duration was 67.9 ± 44.9 months. Compared to patients in R0 group, those in R1 group had a higher percentage of tumor occurrence in the lower extremities (94.3% vs. 70.6%, *p* = 0.031). Detailed information about tumor histopathology and grade of the study population is listed in Table 2. Liposarcoma (25%, 13/52) was the most predominant one, and only 2 were low-grade liposarcoma.

**Table 1:** Patient characteristics

Characteristics	Total (*n* = 52)	R1 (*n* = 35)	R0 (*n* = 17)	*P* value
**Age (mean, years)**	51.0 ± 18.7	51.9 ± 17.3	49.2 ± 21.9	0.641
**Gender**				0.769
Male	28 (53.8)	18 (51.4)	10 (58.8)	
Female	24 (46.2)	17 (48.6)	7 (41.2)	
**Duration of follow-up (month)**	67.9 ± 44.9	62.6 ± 41.6	78.8 ± 50.6	0.226
**Adjuvant chemotherapy**	4 (7.7)	2 (5.7)	2 (11.8)	0.589^a^
**Size (diameter)**				1.000^a^
≤ 5 cm	5 (9.6)	3 (8.6)	2 (11.8)	
> 5 cm	47 (90.4)	32 (91.4)	15 (88.2)	
**Location (extremity)**				**0.031**^a^
Upper	7 (13.5)	2 (5.7)	5 (29.4)	
Lower	45 (86.5)	33 (94.3)	12 (70.6)	
**FNCLCC Grade**				0.096
Grade I	15 (20.6)	13 (37.1)	2 (11.8)	
Grade II	21 (40.4)	11 (31.4)	10 (58.8)	
Grade III	16 (30.8)	11 (31.4)	5 (29.4)	
**Local recurrence**	10 (19.2)	4 (11.4)	6 (35.3)	0.062^a^
**Distant recurrence**	21 (40.4)	14 (40.0)	7 (41.2)	0.935

*Abbreviations*: *R0* microscopically negative margin, *R1* microscopically positive margin, *FNCLCC* the French Federation of Cancer Centers Sarcoma Group

*P*-value < 0.05 is shown in bold

^a^The variable was test by Fisher Exact test

**Table 2:** Histopathology and FNCLCC grade

Histopathlogy	Total number	R0 resection		R1 resection	
		FNCLCC	Number	FNCLCC	Number
Liposarcoma	13				
Low-grade liposarcoma	2			I	2
Myxoid liposarcoma	9	I	1	I	6
		II	1	II	1
Pleomorphic liposarcoma	2			II	1
		III	1		
Leiomyosarcoma	6	II	1	II	1
				III	4
Rhabdomyosarcoma	3	II	1		
		III	1	III	1
Myxofibrosarcoma	7	II	1	I	2
				II	2
				III	2
Malignant peripheral nerve sheath tumor	5			I	2
		II	2	II	1
Synovial sarcoma	5	II	1	II	2
				III	2
Malignant fibrous histiocytoma	2	I	1	II	1
Extraskeletal osteosarcoma	1	III	1		
PNET/Ewing sarcoma	2	II	1	III	1
Spindle cell sarcoma	1			II	1
Alveolar soft part sarcoma	1			II	1
Sarcoma, high-grade	2	II	1		
		III	1		
Myofibroblastic sarcoma	1	II	1		
Undifferentiated pleomorphic sarcoma	2	III	1	III	1
Extraskeletal myxoid chondrosarcoma	1			I	1

*Abbreviation*: *PNET* Primitive neuro-ectodermal tumors

Univariate and multivariable survival analyses were preformed to evaluate the association between local recurrence and resection margin (Table 3). In univariate analysis, FNCLCC Grade had a significant association with local recurrence (Grade III vs I, HR: 10.95, 95% CI: 1.29—92.63, *p* = 0.028). No significant difference in risk for local recurrence was displayed in R1 compared to R0 (HR: 0.39, 95% CI = 0.11- 1.40, *p *= 0.151). After adjusting for FNCLCC Grade, there was still no significance observed between R0 and R1 groups (adjusted HR (aHR): 0.59, 95% CI: 0.15–2.30, *p* = 0.444).

**Table 3:** Univariate and multivariable survival analyses for local recurrence

Variable	Univariate			Multivariable		
	**HR**	**95% CI**	***P*-value**	**aHR**	**95% CI**	*P*-value
Margins (R1 vs R0)	0.39	0.11—1.40	0.151	0.59	0.15—2.30	0.444
Age	1.01	0.97—1.05	0.638			
Gender (Female vs Male)	0.46	0.12—1.79	0.262			
Size (diameter) (> 5 cm vs ≤ 5 cm)	NA	NA	NA			
Location (extremity) (Lower vs Upper)	0.46	0.10—2.16	0.322			
FNCLCC Grade (vs I)						
Grade II	2.24	0.23—21.57	0.484	1.77	0.17—18.62	0.634
Grade III	10.95	1.29—92.63	**0.028**	8.41	0.90—78.76	0.062
Chemotherapy (Yes vs No)	1.62	0.20—12.93	0.650			

*Abbreviations*: *R0* microscopically negative margin, *R1* microscopically positive margin, *FNCLCC* the French Federation of Cancer Centers Sarcoma Group, *NA* no event occurred in a classified subgroup, *HR* hazard ratio, *aHR* adjusted hazard ratio, *CI* confidence interval

*P*-value < 0.05 is shown in bold

Any variable whose univariate analysis had a *p*-value < 0.15 was input as a candidate for the multivariable analysis

**Fig. 1:**
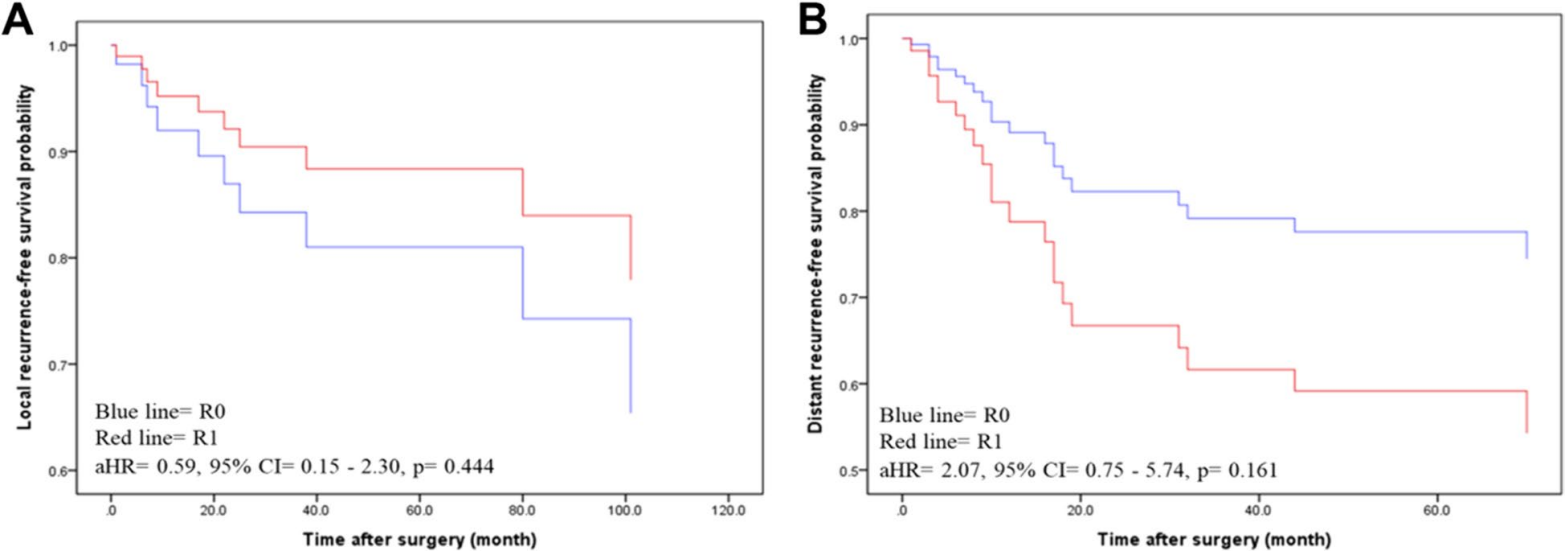
Kaplan–Meier curves display the estimate survival of (**A**) local recurrence-free survival and (**B**) distant recurrence-free survival according to resection margin status. R0, microscopically negative margin; R1, microscopically positive margin; aHR, adjusted hazard ratio; CI, confidence interval

Univariate and multivariable survival analyses were used to evaluate the association between distant recurrence and resection margin (Table 4). In univariate analysis, FNCLCC Grade had a significant association with distant recurrence (Grade III vs I, HR: 9.48, 95% CI: 2.08–43.24, *p* = 0.004). No significant difference was displayed in risk for local recurrence in R1 compared to R0 (HR: 1.07, 95% CI: 0.43–2.66, *p* = 0.882). After adjusting for location and FNCLCC Grade, there was still no significance observed between R0 and R1 groups (aHR: 0.59, 95% CI: 0.15–2.30, *p* = 0.444). However, tumor location in lower extremities (Lower vs upper, aHR: 0.23, 95% CI: 0.07–0.70, *p* = 0.010) and FNCLCC Grade (Grade III vs I, aHR: 12.53, 95% CI: 2.67–58.88, *p* = 0.001) had significant association with distant recurrence. The Kaplan–Meier plots showed that no significant differences in local recurrence-free survival (*p* = 0.444) and distant recurrence-free survival (*p* = 0.161) for 5 years were observed between R1 and R0 groups (Fig. 1).

**Table 4:** Univariate and multivariable survival analyses for distant recurrence

Variable	Univariate			Multivariable		
	**HR**	**95% CI**	***P-va*lue**	**aHR**	**95% CI**	***P*-value**
Margins (R1 vs R0)	1.07	0.43—2.66	0.882	2.07	0.75—5.74	0.161
Age	1.02	0.99—1.04	0.215			
Gender (Female vs Male)	0.65	0.27—1.57	0.340			
Size (diameter) (> 5 cm vs ≤ 5 cm)	NA	NA	NA			
Location (extremity) (Lower vs Upper)	0.39	0.14—1.06	0.066	0.23	0.07—0.70	**0.010**
FNCLCC Grade (vs I)						
Grade II	3.38	0.72—15.91	0.124	3.55	0.71—17.91	0.124
Grade III	9.48	2.08—43.24	**0.004**	12.53	2.67—58.88	**0.001**
Chemotherapy (Yes vs No)	2.25	0.66—7.67	0.194			

*Abbreviations: R0 *microscopically negative margin*, R1 *microscopically positive margin*, FNCLCC *the French Federation of Cancer Centers Sarcoma Group*, NA *no event occurred in a classified subgroup*, HR *hazard ratio*, aHR *adjusted hazard ratio*, CI *confidence interval

*P*-value < 0.05 is shown in bold

Any variable whose univariate analysis had a *p*-value < 0.15 was input as a candidate for the multivariable analysis

## Discussion

The present study showed that local or distant recurrence-free survivals of R1 resection, when completed by radiotherapy, are not inferior to R0 resection in patients with localized ESTS. Histological FNCLCC grade III and tumor location are associated with distant recurrence.

The results showed that R0/R1 resection margin is not associated with local or distant recurrence of localized ESTS, which challenges the traditional emphasis on R0 resection. Similarly, several studies reported negative margin is not an independent factor of survivals [11–14]. Kim et al. reported no association between local recurrence and positive or close resection margin when adjuvant radiotherapy was used in 150 patients with extremity or truncal STS, but R0 resection group had better distant metastasis-free survival [11]. Harati et al. reported comparable survivals between R0 and R1 margins in 164 patients with leiomyosarcoma [12] and 120 patients with ESTS [13], in which postoperative radiotherapy was not associated with survival. Olson et al. also reported similar local recurrence-free survival between R0 and R1 resection in 97 patients with well-differentiated liposarcoma [14]. Noticeably, another retrospective study by Harati et al. focusing on 110 patients with chest wall STS underwent surgical treatment reported that although patients with R0 margins had better 5-year overall survival compared to positive margins, R0 margin was not associated with survival in multivariate analysis [17]. The authors proposed that positive resection margin was more frequent in patients with high histological grade, therefore it may be a result rather than a cause of tumor aggressiveness.

The role of adjuvant radiotherapy in the treatment of localized STS remains clarification. Several recent studies discussed the impact of quantitative width of the negative margin with adjuvant radiotherapy. Ahmad et al. evaluated the correlation between quantitative resection margin width and outcomes in 382 patients with localized extremity or truncal STS who underwent limb-sparing surgery and radiotherapy [9]. They reported no significant differences in local recurrence rate or survival in the groups with a negative margin of width of ≤ 1 mm, > 1 mm, ≤ 5 mm, and > 5 mm. With adjuvant radiotherapy, the quantitative width of the negative margin does not influence the outcome, and it is unnecessary to attempt a wider negative resection margin. Harati et al. also reported that close and wide negative margins led to similar local recurrence-free survival in 643 patients with primary ESTS among categorized (1 mm vs. 1–5 mm vs. > 5 mm) negative margins, while 270 patients (42%) with adjuvant radiotherapy had similar local outcomes with different widths of the negative margin [18]. A systemic review by Strander et al. showed that the combination of limb-sparing surgery and adjuvant radiotherapy had a 90% local control rate in patients with STS of extremities and trunk who underwent resection with negative, marginal, or minimal microscopic positive surgical margins [4]. Taken together, surgical resection should achieve R0 for the low local recurrence when the condition is feasible. If tumors are near critical neurovascular structures, bones, or joints, planned resection with close margin plus postoperative radiotherapy may also achieve good local control.

Our study showed that high FNCLCC grade is associated with distal recurrence. The incidence of distant recurrence is about 25% in patients with ESTS [1, 4]. Pulmonary metastasis is the most common form of metastatic disease. Risk factors for distant recurrence are tumor profiles including tumor size, grade, and histologic subtype, showing that tumor-related factors are more important than treatment-related factors to distant recurrence [19–24].

## Limitations

The study has several limitations. First, the small sample size is the critical limitation to draw robust conclusions. Second, more caution in data interpretation and generalization is needed because of the predominance of liposarcomas in the sample. Third, it lacks a non-radiotherapy control group to clarify the role of radiotherapy in the outcomes of R0/R1 resection. Fourth, it has the limitations inherent to the retrospective nature.

## Conclusions

The present study showed R1 margins, when complemented by radiotherapy, did not significantly alter outcomes of ESTs as in R0 margin. Histological FNCLCC grade and primary tumor location are associated with distant recurrence. Further studies involving more diverse sarcoma types and larger cohorts are necessary to better highlight the path forward and confirm the results in broader clinical contexts.

## Data Availability

No datasets were generated or analysed during the current study.

## Abbreviations

ESTS: Extremity soft tissue sarcoma
UICC: Union for International Cancer Control
3DCRT: Three-dimensional conformal radiotherapy
IMRT: Intensity modulated radiotherapy
VMAT: Volumetric modulated arc therapy
SD: Standard deviation
HRs: Hazard ratios
Cis: Confidence intervals
